# The Medico-Legal Society of Chicago

**Published:** 1886-06

**Authors:** 


THE MEDICO-LEGAL SOCIETY OF CHICAGO.

Attention was called in the May number of the JOURNAL to the efforts of Dr. A. Reeves Jackson, Dr. Edward J. Doering and others in the establishment of a "Physicians' Protective Association."

On the fifteenth of May, 1886, an organization was effected, the association being termed the "MEDICO-LEGAL SOCIETY OF CHICAGO."

The object of the SOCIETY, as specified in the Constitution, is:

"The investigation, study and advancement of the science of medical jurisprudence, the punishment of unprofessional and criminal practice by members of the medical and legal professions, the prevention of blackmailing, and the procuring of such legislation as may be necessary to secure the ends above enumerated."

"Regular meetings shall be held on the first Saturday in September, December, March and June, and ten members shall constitute a quorum."

Over one hundred names have already been enrolled upon the list of members, and the profession is keenly alive to the importance of the organization. The adoption of the title, "Medico-Legal Society," obviates at once many of the objections, urged with force, against a PHYSICIANS' PROTECTIVE ASSOCIATION.

